# Multimodal *α*-Glucosidase and *α*-Amylase Inhibition and Antioxidant Effect of the Aqueous and Methanol Extracts from the Trunk Bark of *Ceiba pentandra*

**DOI:** 10.1155/2020/3063674

**Published:** 2020-04-20

**Authors:** Telesphore Benoit Nguelefack, Christian Kuete Fofie, Elvine Pami Nguelefack-Mbuyo, Adeline Kaptue Wuyt

**Affiliations:** Laboratory of Animal Physiology and Phytopharmacology, Department of Animal Biology, Faculty of Science, University of Dschang, P.O. Box 67, Dschang, Cameroon

## Abstract

Postprandial hyperglycemia and oxidative stress are important factors that worsen the health condition of patients with type 2 diabetes. We recently showed that extracts from *Ceiba pentandra* mitigate hyperglycemia in dexamethasone- and high diet/streptozotocin-induced diabetes. Herein, we evaluated the postprandial regulatory properties and the antioxidant effects of the aqueous (AE) and methanol (ME) extracts from the stem bark of *Ceiba pentandra*. The phytochemical analysis of AE and ME was performed using the LC-MS technique and the total phenolic and flavonoid assays. Both extracts were tested for their ability to inhibit superoxide anion (O_2_^•ـ^), hydrogen peroxide (H_2_O_2_), protein oxidation, alpha-amylase, and alpha-glucosidase activities. The mode of enzyme inhibition was also determined in a kinetic study. AE and ME were both rich in phenolic and flavonoid compounds. ME was 2.13 and 1.91 times more concentrated than AE in phenolic and flavonoid compounds, respectively. LC-MS allowed the identification of 5 compounds in both extracts. ME and AE inhibited O_2_^•ـ^ with IC_50_ of 51.81 and 34.26 *μ*g/ml, respectively. On H_2_O_2_, they exhibited IC_50_ of 44.84 and 1.78 *μ*g/ml, respectively. Finally, they exhibited IC_50_ of 120.60 and 140.40 *μ*g/ml, respectively, in the inhibition of protein oxidation induced by H_2_O_2_, while showing IC_50_ of 39.26 and 97.95 *μ*g/ml on the protein oxidation induced by AAPH. ME and AE inhibited alpha-amylase with IC_50_ of 6.15 and 54.52 *μ*g/ml, respectively. These extracts also inhibited alpha-glucosidase, demonstrating IC_50_ of 76.61 and 86.49 *μ*g/ml. AE exhibited a mixed noncompetitive inhibition on both enzymes, whereas ME exhibited a competitive inhibition on *α*-amylase and a pure noncompetitive inhibition on *α*-glucosidase. These results demonstrate that ME and AE scavenge reactive oxygen species and prevent their effects on biomolecules. Besides, ME and AE inhibit carbohydrate digestive enzymes. These properties may contribute to reduce postprandial hyperglycemia and regulate glycemia in diabetic patients.

## 1. Introduction

Diabetes mellitus is becoming a serious and leading health threat in low- and middle-income countries. It is one of the largest global health emergencies of the 21st century with increasing prevalence. Approximately 463 million adults aged 20 to 79 years are currently living with diabetes, and by 2045, this will rise to 700 million. Diabetes accounted for 4.2 million deaths worldwide in 2019 [1]. Chronic hyperglycemia is a common feature in all diabetic patients. Therefore, the main strategy in the management of diabetes is the achievement of an adequate glycemic control. Fasting glycemia is routinely measured as a determinant of glycemic control, but although necessary, it is insufficient. To achieve an effective blood glucose control, postprandial glycemia should also be taken into consideration. In fact, the loss of postprandial glycemic control has been shown to be an early step of glucose homeostasis disorder that occurs earlier than fasting glycemia impairment [2, 3]. Recent evidences have demonstrated that postprandial hyperglycemia is an independent risk factor for both microvascular and macrovascular complications in both type 1 and type 2 diabetes mellitus [4, 5]. Micro- and macrovascular changes are a preeminent cause of diabetes-related death and disability [6].

Besides, hyperglycemia has been associated with excess free radical production [7] which results in oxidative stress (OS). OS is a key component in diabetic complications acting through various mechanisms including increased flux of the polyol pathway, advanced glycation end product formation and accumulation, overactivation of the hexosamine pathway, and protein kinase C activation [8, 9].

One current approach in the management of postprandial hyperglycemia consists in slowing down the absorption of carbohydrates using inhibitors of digestive enzymes such as acarbose, voglibose, and miglitol. Although these drugs have beneficial effects such as weight loss, they have unpleasant gastrointestinal side effects that frequently result in therapy abandonment [10]. The search for other therapeutic alternatives is therefore encouraged, especially those which can address both postprandial hyperglycemia and oxidative stress.

Plants are important sources of antioxidants. Hydroxytyrosol, for example, a phenolic compound obtained from the olive tree, is currently considered the most powerful antioxidant [11]. Among the groups of secondary metabolites most known for their antioxidant activities, polyphenols and especially flavonoids are of particular interest. Intensive literature has demonstrated their benefits and their structure-related activities [12, 13].

Previous studies performed by our research team showed that *Ceiba pentandra* (L.) Gaertn. stimulates glucose utilization and reduces glucose release by the liver [14], promotes glycogen synthesis and prevents gluconeogenesis [15], exhibits antioxidant activity against DPPH and hydroxyl radical, prevents lipid peroxidation and red blood cell hemolysis [14], and possesses antidiabetic effects on dexamethasone-treated rats [16] and on high-fat diet/streptozotocin-treated rats [17]. Based on these studies, we undertook to quantify the total phenolic and flavonoid contents in the methanol and aqueous extracts of *Ceiba pentandra* trunk bark, to characterize and compare by LC-MS the chemical composition of the aqueous and methanol extracts, to investigate their scavenging activities on other oxidant systems yet unstudied, and to evaluate the effects of these extracts on the carbohydrate digestive enzymes.

## 2. Materials and Methods

### 2.1. Extract Preparation

Fresh barks from the trunk of *Ceiba pentandra* were harvested in Yaoundé (center region of Cameroon) in 2016 by a botanist, Dr. Tsabang Nolé, and authenticated at the Cameroon National Herbarium by comparison with an existing specimen no. HNC 43623. The barks were air dried, and the dry barks were powdered using a grinder. Two hundred grams (200 g) of the obtained powder were boiled for 20 minutes in 2 l distilled water. The filtrate was freeze dried and yielded 8.86 g of aqueous extract that was stored at 2°C until use.

The methanol extract was prepared as follows: 200 grams of powder were macerated in 2 l of methanol for 48 hours. The filtrate was concentrated on a rotary evaporator. After collection, the extract was put in an oven at 40°C for 24 h to remove residual methanol. The resulting 3.9 g of pellets representing the methanol extract was stored at 2°C until use.

### 2.2. Chemicals

Folin-Ciocalteu's reagent, sodium carbonate, aluminum chloride, sodium nitrate, potassium hydroxide, methanol, gentamycin, ethylene diamine tetra-acetic acid, sodium hydroxide, gallic acid, quercetin, sodium hydrogen phosphate, sodium dihydrogen phosphate, trichloroacetic acid, nitroblue tetrazolium, Tris, nicotinamide adenine dinucleotide, phenazine methosulfate, hydrogen peroxide, ascorbic acid, bovine serum albumin (BSA), Coomassie blue, sodium chloride, 2′,2′-azobis (2-methylpropionamidine) dihydrochloride (AAPH), 5,5′-dithiobis (2-nitrobenzoic acid) acid, potassium cyanide, *α*-amylase, *α*-glucosidase, dinitrosalicylic acid, starch, p-nitrophenyl glucopyranoside, glucose, and dimethyl sulfoxide were all purchased from Sigma-Aldrich Chemical Co. (Taufkirchen, Germany).

### 2.3. Experimental Protocols

#### 2.3.1. Determination of Total Phenolic Content

The amount of total phenols in the extracts was determined spectrophotometrically by the Folin-Ciocalteu reagent method [18]. Optical density was read at 750 nm. The concentration of total phenols in the extracts was determined from the gallic acid standard curve and expressed as milligrams of gallic acid equivalent per 100 g of dry mass extract (mg EAG/100 g). The experiment was done in triplicate.

#### 2.3.2. Determination of Total Flavonoids (TF)

Colorimetric determination of total flavonoid content in *C. pentandra* extracts was performed using aluminum chloride as described by Chang et al. [19] with quercetin as a standard. Optical density was read at 510 nm. The concentration of total flavonoids in the extracts was determined from the quercetin standard curve and expressed in milligrams of quercetin equivalent per 100 g of dry mass of extract (mg EQ/100 g). The assay was done in triplicate.

#### 2.3.3. LC-MS Analysis

The following parameters were used for the LC-MS analysis: spray voltage of 4.5 kV and capillary temperature of 200°C. Nitrogen was used as sheath gas (10 l/min). The spectrometer was attached to an UltiMate 3000 UHPLC System (Thermo Fisher Scientific, USA) consisting of an LC pump, diode array detector (DAD) (*λ*: 190-600 nm), autosampler (injection volume 10 *μ*l), and column oven (40°C). The separations were performed using a Synergi MAX-RP 100A (50 × 2 mm, 2.5 *μ*m particle size) with a H_2_O (+0.1% HCOOH) (A)/acetonitrile (+0.1% HCOOH) (B) gradient (flow rate 500 *μ*l/min, injection volume 10 *μ*l). Samples were analyzed using a gradient program as follows: 95% A isocratic for 1.5 min and linear gradient to 100% B over 6 min, and after 100% B isocratic for 2 min, the system returned to its initial condition (90% A) within 1 min and was equilibrated for 1 min. High-resolution mass spectra were obtained with a QTOF Spectrometer (Bruker, Germany) equipped with a HESI source. The spectrometer was operated in positive mode (mass range: 100-1500, with a scan rate of 1.00 Hz) with automatic gain control to provide high-accuracy mass measurements within 0.40 ppm deviation using Na formate as calibrant.

#### 2.3.4. Superoxide Scavenging Test

The radical scavenging activity of the aqueous and methanol extracts from *C. pentandra* trunk bark was assessed on superoxide anion using a method described by Robak and Gryglewski [20] with some modifications. Superoxide anions were generated in a phenazine methosulfate- (PMS-) NADH system. The reaction mixture was made of 0.5 ml test solution, 0.95 ml 0.1 M phosphate buffer (pH 7.4), 0.5 ml 20 mM PMS, 156 mM NADH, and 25 mM NBT in phosphate buffer (pH 7.4). Extracts were used at concentrations of 1, 3, 10, 30, 100, and 300 *μ*g/ml. Gallic acid was tested at the same concentrations and used as a reference drug. Reduction of nitroblue tetrazolium was monitored at 560 nm on a Helios Epsilon Spectrophotometer (Thermo Fisher Scientific). The test was carried out in triplicate. The percent inhibition was calculated as follows:

(1) $$\begin{array}{c} \%\text{inhibition}=100\times \frac{\text{OD control}-\text{OD sample}}{\text{OD control}}. \end{array}$$

#### 2.3.5. Hydrogen Peroxide Scavenging Activity

A method previously described by Ruch et al. [21] was used to assess the ability of plant extracts to decompose hydrogen peroxide (H_2_O_2_). Briefly, a 40 mM solution of H_2_O_2_ prepared in phosphate buffer (pH 7.4) was mixed with graded concentrations (1-300 *μ*g/ml) of extracts or ascorbic acid. After 10 min of incubation, optical densities were read at 230 nm against a blank solution made up of phosphate buffer without H_2_O_2_. The percentage of H_2_O_2_ scavenging activity was calculated as in the previous test.

#### 2.3.6. H_2_O_2_- and AAPH-Induced Protein Oxidation Assay

The effect of plant extracts against protein oxidation was evaluated according to the method of Simplicio et al. [22] with some modifications. Zero point five milliliter aliquots of BSA (30 mg/ml) prepared in phosphate buffer saline (50 mM, pH 7.4) were incubated with 0.5 ml of extracts or gallic acid. Fifteen minutes later, 0.5 ml of H_2_O_2_ (20 mM) or AAPH (2′,2′-azobis (2-methylpropionamidine) dihydrochloride) solution (50 mM) in another set of experiment, was added to the reaction medium and incubated at 37°C for 30 minutes and 1 hour, respectively. Proteins were precipitated with ammonium sulfate (70%) followed by vigorous shaking and centrifugation at 3000 rpm. The pellet obtained was resuspended in 1.5 ml of PBS, and 0.2 ml of Ellman's reagent was introduced. The level of thiol groups was measured 10 minutes later at 412 nm.

#### 2.3.7. *α*-Amylase Inhibitory Test

The alpha-amylase inhibitory test was performed using a modified procedure of McCue and Shetty [23]. A volume of 250 *μ*l of extract or acarbose (1-300 mg/ml) was mixed with 250 *μ*l of 0.02 M sodium phosphate buffer (pH 6.9) containing *α*-amylase at a concentration of 0.5 mg/ml. The mixture was preincubated at 25°C for 10 minutes. Then, 250 *μ*l of 1% starch solution in 0.02 M sodium phosphate buffer (pH 6.9) was added and incubated at 25°C for another 10 minutes. The reaction was stopped by adding 500 *μ*l of dinitrosalicylic acid (DNS). The tubes were then incubated in a water bath at 95°C for 5 minutes and cooled at room temperature followed by dilution with 5 ml distilled water. The optical density was measured at 540 nm. The inhibitory activity on alpha-amylase was calculated as percent inhibition using the following formula:

(2) $$\begin{array}{c} \%\text{inhibition}=\left(\frac{\text{OD control}-\text{OD extracts}}{\text{OD control}}\right)\times 100. \end{array}$$

#### 2.3.8. Determination of the Inhibitory Mode of Extracts on *α*-Amylase

To determine the mode of inhibition of the plant extracts on the activity of alpha-amylase, the kinetic of action of these extracts was evaluated using three concentrations: IC_50_/2, IC_50_, and IC_50_ × 2. The method used was a modification of that described by Ali et al. [24]. Extract solution (250 *μ*l) was preincubated with 250 *μ*l of *α*-amylase solution (7.5 U/ml) for 10 minutes at 25°C. Two hundred and fifty microliters (250 *μ*l) of starch solution at increasing concentrations (3, 6, 9, 12, and 15 mg/ml) were added to start the reaction. The reaction mixture was incubated for 10 minutes at 25°C and then at 95°C for 5 minutes after the addition of 500 *μ*l DNS to stop the reaction. In control tubes, extracts were replaced by phosphate buffer (pH 6.9). Optical densities were red at 540 nm and converted into reaction rates. A double reciprocal graph (1/*v* versus 1/(*S*)), where *v* is the reaction rate and (*S*) the concentration of the substrate, was plotted. The mode of inhibition of the extract on *α*-amylase activity was determined using the Lineweaver-Burk curve [25].

#### 2.3.9. *α*-Glucosidase Inhibitory Test

The ability of *C. pentandra* extracts to inhibit the activity of *α*-glucosidase was assessed according to Kim et al.'s [26] protocol. Shortly, *α*-glucosidase (1 U/ml) from *Saccharomyces cerevisiae* was preincubated with 250 *μ*l extracts for 10 minutes. P-nitrophenyl glucopyranoside substrate solution (pNPG, 3 mM) prepared in 20 mM phosphate buffer (pH 6.9) containing 2 mg/ml BSA was added to start the reaction. The reaction mixture was incubated at 37°C for 20 minutes and stopped with 1 ml of Na_2_CO_3_ (1 M). *α*-Glucosidase activity was determined by measuring paranitrophenol released from pNPG at 405 nm. The percent inhibition was calculated as follows:

(3) $$\begin{array}{c} \%\text{inhibition}=\left(\frac{\left(\text{OD control}-\text{OD sample}\right)}{\text{OD control}}\right)\times 100. \end{array}$$

#### 2.3.10. Determination of the Inhibitory Mode of Extracts on *α*-Glucosidase

Three different concentrations of extracts, namely, IC_50_/2, IC_50_, and IC_50_ × 2, were used to determine the mode of inhibition of *α*-glucosidase by *C. pentandra* extracts. A modified method of Ali et al. [24] in 2006 was used. Indeed, different extract concentrations at 250 *μ*l each were preincubated with 500 *μ*l of *α*-glucosidase solution for 10 minutes at 25°C. Then, 250 *μ*l of pNPG substrate at increasing concentrations (1.25, 2.5, 5, 10, and 20 mM) were added to the reaction mixture. This reaction mixture was further incubated for 10 minutes at 25°C, and Na_2_CO_3_ was added to stop the reaction. The optical densities were measured and converted to reaction rate. The mode of inhibition was determined using the Lineweaver-Burk curve as previously described.

#### 2.3.11. Statistical Analyses

Data are expressed at mean ± standard error of the mean. IC_50_ values were obtained after logarithmic transformation of the concentration-response curve using GraphPad Prism Software 5.01. The one-way analysis of variance (ANOVA) followed by the posttest of Tukey was used to analyze the data from total phenol and flavonoid content. The nonlinear regression (curve fit) with the sigmoidal dose-response equation was used to analyze data from all the other tests except for the enzyme kinetics, and the best fit parameters (logIC_50_ and top) were compared. Differences were considered significant when the probability threshold *p* was less than 0.05. Where necessary, the efficiency index was calculated using the following formula [14]:

(4) $$\begin{array}{c} \text{Efficiency index}=\frac{E_{\max}}{\text{I}{\text{C}}_{50}}. \end{array}$$

## 3. Results

### 3.1. Phenolic and Flavonoid Content in the Extracts

**Figure 1:**
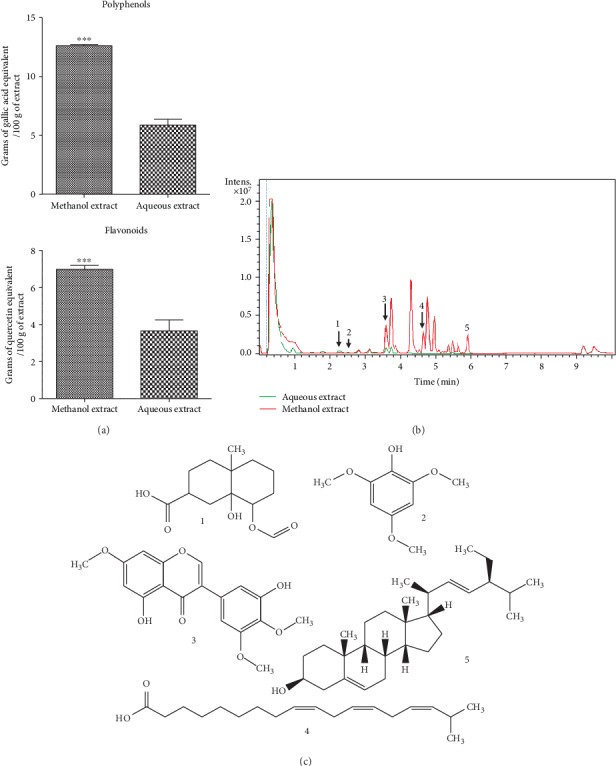
Phytochemical analysis of the aqueous and methanol extracts of the stem bark of *Ceiba pentandra*. (a) Polyphenol and flavonoid contents in the aqueous and methanol extracts of *C. pentandra* (*n* = 3). (b) LC fingerprint of extracts detected with UV 190-600 nm. (c) Identified compounds (**1**-**5**) are indicated by peak numbers on the fingerprint. **1**: 8-(formyloxy)-8a-hydroxy-4a-methyldecahydro-2-naphthalene carboxylic acid; **2**: 2,4,6-trimethoxyphenol; **3**: 5,3′-dihydroxy-7,4′,5′-trimethoxyisoflavone; **4**: 17-hydroxlinoleic acid; **5**: stigmasterol. ^∗∗∗^*p* < 0.001 represents significant difference with respect to the aqueous extract.

The results presenting the total phenolic and flavonoid content in *C. pentandra* extracts are shown in Figure 1(a). Regardless of the type of secondary metabolite evaluated, the methanol extract was always richer than the aqueous extract. The phenolic content was 2.13 times significantly (*p* < 0.001) higher in the methanol extract (12.62 ± 0.12 EAG/100 g) than in the aqueous extract (5.88 ± 0.51 EAG/100 g). Similarly, the flavonoid content in the methanol extract (6.99 ± 0.21 EQ/100 g) was 1.91 times significantly (*p* < 0.01) higher than in the aqueous extract (3.66 ± 0.60 EQ/100 g).

### 3.2. LS-MS Phytochemical Analysis

The fingerprint of the LC presented in Figure 1(b) shows common peaks in AE and ME as well as peaks only present in each of the extracts. The combination of data from the literature and information from the MS spectra allows tentative identification of a total of five compounds presented in Figure 1(c). Compounds **1** and **3** were present in both extracts. Compound **2** was only present in AE, while compounds **4** and **5** were visible only in ME. Compound **1** appears at RT 2.3 min with [M + H]^+^ at *m*/*z* 257 and was identified as 8-(formyloxy)-8a-hydroxy-4a-methyldecahydro-2-naphthalene carboxylic acid [27, 28]. Compound **2** (RT 2.6 min) showed [M + H]^+^ at *m*/*z* 185 and was identified as 2,4,6-trimethoxyphenol [29, 30]. Compound **3** appears at RT 3.6 min with [M + H]^+^ at *m*/*z* 345 and was identified as 5,3′-dihydroxy-7,4′,5′-trimethoxyisoflavone or vavain [31, 32]. Compound **4** (RT 4.7 min) showed [M + H]^+^ at *m*/*z* 295 and was identified as 17-hydroxlinoleic acid [33, 34]. Compound **5** showed a peak at 5.9 min, [M + H]^+^ at *m*/*z* 413 and was identified as stigmasterol [34].

### 3.3. Superoxide Anion Radical Scavenging Activity

**Figure 2:**
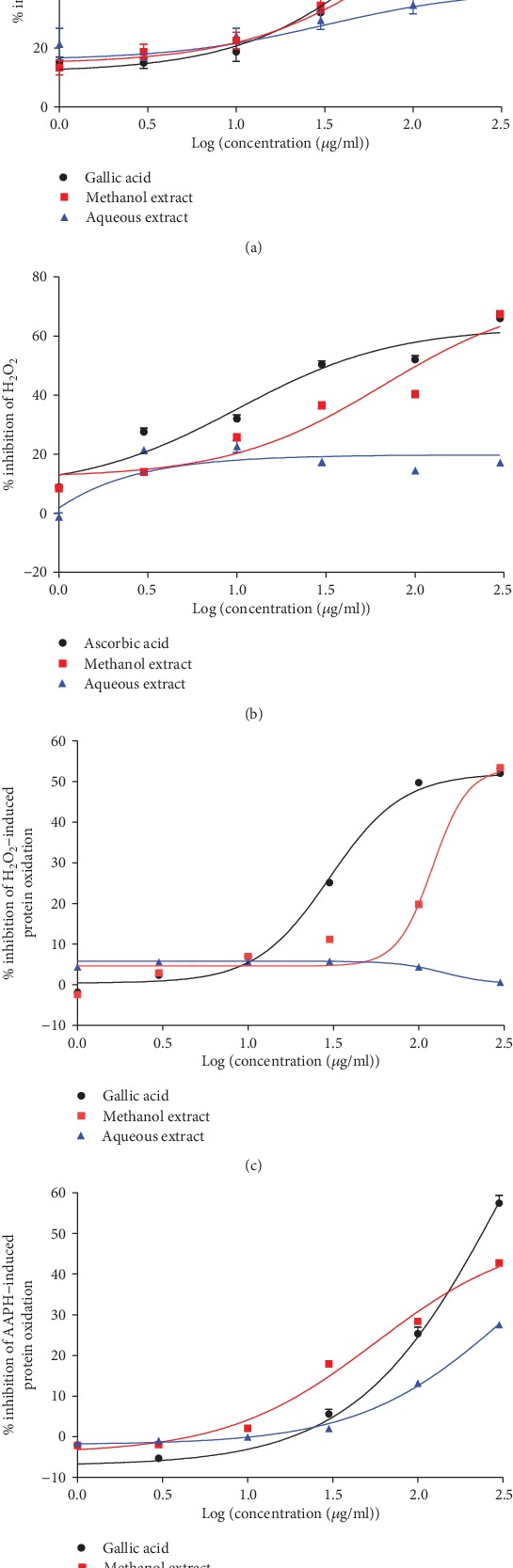
*In vitro* antioxidant effects of the aqueous and methanol extracts of the stem bark of *Ceiba pentandra*. (a) Inhibitory effect of extracts on superoxide anion. (b) Inhibitory effect of extracts on hydrogen peroxide. (c) Inhibitory effect of extracts on protein oxidation by hydrogen peroxide. (d) Inhibitory effect of extracts on protein oxidation by AAPH. Each point is the mean ± SEM of 5 repetitions.

As depicted in Figure 2(a), both the aqueous and the methanol extracts from the trunk bark of *C. pentandra* exhibited a concentration-dependent radical scavenging activity on superoxide anion. A significant difference (*p* < 0.03) was observed between the activities of the tested substances. With respect to the efficiency index (Table 1), gallic acid was the most effective compound followed by the methanol extract and the aqueous extract.

### 3.4. Hydrogen Peroxide Radical Scavenging Activity of *Ceiba pentandra* Extracts

The results obtained from this test showed that only the ascorbic acid and the methanol extract were capable of scavenging H_2_O_2_ (Figure 2(b)). The aqueous extract was a poor H_2_O_2_ scavenger with an *E*_max_ of 22.64% produced at a concentration of 10 *μ*g/ml. Considering IC_50_ and the efficiency index (*E*_max_/IC_50_), the best efficiency was attributed to ascorbic acid that was significantly (*p* < 0.005) effective than the methanol and the aqueous extracts (Table 1).

### 3.5. Inhibitory Effect of *Ceiba pentandra* against Hydrogen Peroxide-Induced Protein Oxidation

The ability of the extracts to prevent the oxidation of proteins induced by hydrogen peroxide is shown in Figure 2(c). It can be seen that, similar to the H_2_O_2_ scavenging test, the aqueous extract exhibited almost no antioxidant activity against H_2_O_2_-induced protein oxidation. Although the methanol extract had the same maximum effect as that of gallic acid, the latter was shown to be significantly (*p* < 0.0001) and four times more potent than the methanol extract (Table 1).

### 3.6. Inhibitory Effect of *Ceiba pentandra* against AAPH-Induced Protein Oxidation

As depicted in Figure 2(d), all tested substances exhibited a concentration-dependent inhibitory effect against peroxyl radical- (AAPH-) induced protein oxidation. The best plant extract activity was obtained with the methanol extract, which was found to be more potent (*p* < 0.0001) than gallic acid, used in this experiment as the reference drug (Table 1).

### 3.7. Inhibitory Effect of *Ceiba pentandra* Extracts on the Activity of Alpha-Amylase

**Figure 3:**
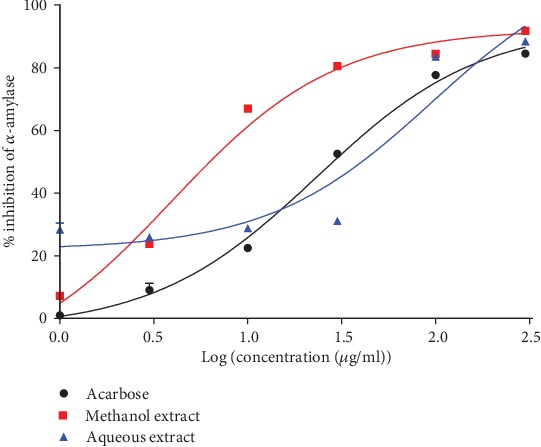
Aqueous and methanol extracts of the stem bark of *Ceiba pentandra* concentration-dependently inhibit *α*-amylase activity. Each point represents the mean + standard error of 5 repetitions.

It can be observed from Figure 3 that all tested substances substantially inhibited *α*-amylase activity in a concentration-dependent manner. At the highest concentrations used, inhibition percentages produced by both extracts were nearly the same as that of acarbose. Maximum inhibitions were 84%, 91%, and 88%, respectively, for acarbose, methanol extract, and aqueous extract. Considering both IC_50_ and EI (Table 1), it appears that the methanol extract (EI = 14.93) was approximately 4 times (*p* < 0.001) more active than acarbose (EI = 4.03) and about 9 times (*p* < 0.0001) more active than the aqueous extract (EI = 1.63).

**Figure 4:**
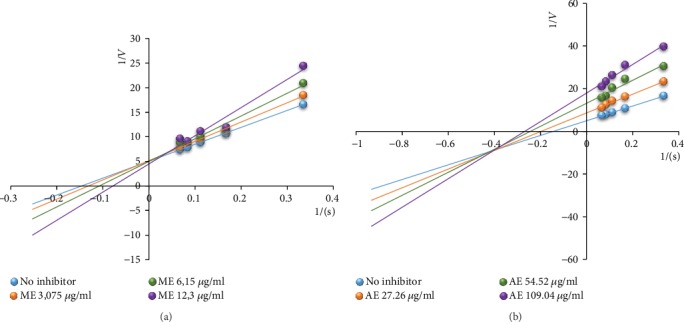
Inhibition mode of the methanol (a) and aqueous (b) extracts of *C. pentandra* on *α*-amylase. ME: methanol extract; AE: aqueous extract. Each point represents the mean of 5 repetitions.

The evaluation of the mode of inhibition shows that the methanol extract exhibited a competitive inhibition (Figure 4(a)), while the aqueous extract exerted a mixed noncompetitive inhibition (Figure 4(b)).

### 3.8. Inhibitory Effect of Extracts on the Activity of Alpha-Glucosidase

**Figure 5:**
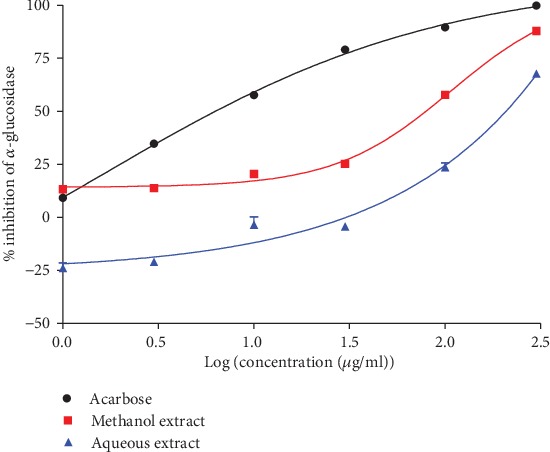
Aqueous and methanol extracts of the stem bark of *Ceiba pentandra* concentration-dependently inhibit *α*-glucosidase activity. Each point represents the mean + standard error of 5 repetitions.

The methanol extract inhibited alpha-glucosidase more effectively than the aqueous extract with a maximum effect of 87.79% at the highest concentration used (300 *μ*g/ml) versus 63.73% for the aqueous extract (Figure 5). Nevertheless, the inhibitory effect of acarbose was (*p* < 0.0001) 10 times greater than that of the methanol extract and more than 15 times the magnitude of the aqueous extract according to the efficacy index (Table 1).

**Figure 6:**
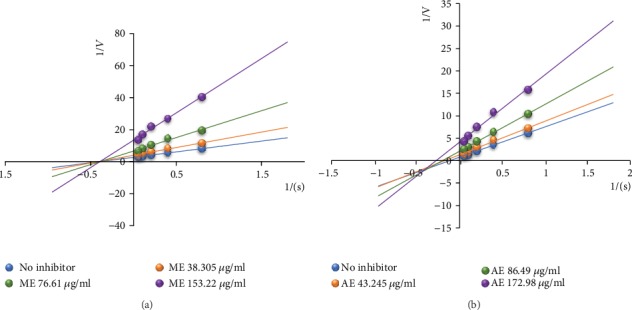
Inhibition mode of the methanol (a) and aqueous (b) extracts of *C. pentandra* on *α*-glucosidase. ME: methanol extract; AE: aqueous extract. Each point represents the mean of 5 repetitions.

The evaluation of the inhibition mode showed that the methanol extract has a pure noncompetitive (Figure 6(a)) mode of action, while the aqueous extract exhibited a mixed noncompetitive inhibition (Figure 6(b)).

## 4. Discussion

Type 2 diabetes is a metabolic disorder culminating in the development of cardiovascular and neurological dysfunctions through the generation of oxidative stress [7]. The present study was designed to get more insight into the antioxidant mechanisms of *C. pentandra* on one hand, and to investigate its ability to prevent postprandial hyperglycemia on the other hand.

Reactive oxygen species (ROS) and mostly superoxide anion are generated from the mitochondrial respiratory chain, and their excessive production may result in oxidative stress. Although superoxide anion is a weak oxidant, it is known as an initial radical and plays an important role in the formation of other ROS, such as hydrogen peroxide [35]. Targeting superoxide appears to be the best way to tackle oxidative stress as this will stop the reaction cascade leading to the formation of other ROS. The present study showed that both extracts and mostly the methanol extract successfully scavenged superoxide anion, suggesting that these plant extracts could be useful tools in the fight against oxidative stress-induced tissue damages.

It is well known that superoxide can spontaneously [36] or enzymatically dismutate into H_2_O_2_ [37]. In a recent study, it has been proven that H_2_O_2_ mediates pathways leading to hyperglycemia via ERK and p38 MAPK in human pancreatic cancer [38]. The ability of *C. pentandra* to scavenge this molecule was evaluated, and it was observed that only the methanol extract exhibited an antioxidant activity against H_2_O_2_. This difference in activity may be due to the difference in composition of both extracts and further demonstrate that compound **1** common to the two extracts and more abundant in the aqueous extract is not responsible for this activity.

In order to effectively verify whether these extracts were able to protect cellular components against the direct deleterious effect of hydrogen peroxide on biological molecules such as proteins, the inhibition of the oxidation of albumin was carried out. Consistent with the previous test, the aqueous extract had no effect, and the methanol extract inhibited by 53% the oxidative action of hydrogen peroxide on albumin. This result suggests that in case of oxidative stress, with overproduction of H_2_O_2_ like that observed in diabetes or cardiovascular diseases, the methanol extract of *C. pentandra* would be an important asset to limit the degradation of cell molecules.

It was then evident that the aqueous extract was unable to protect proteins against oxidation induced by H_2_O_2_ which is generally at the initiation phase; but whether *C. pentandra* extracts could prevent oxidation from free radicals and at the propagation phase is still unknown. Peroxyl radicals (ROO^•^) are the main oxidants responsible from the propagation phase [39] which is also called the amplification phase. Hence, the ability of the plant extracts to inhibit peroxyl radical-induced protein oxidation was investigated using AAPH. The methanol extract of *Ceiba pentandra* inhibited this oxidation by 42% and the aqueous extract by 27%. Although having a moderate activity, the methanol extract was more effective than gallic acid used as reference. This result shows that the methanol extract is an inhibitor of both initiation and propagation of peroxidation reactions, while the aqueous extract has a moderate effect only on the propagation phase. Protein oxidation plays an important role in the etiology of diabetes and cardiovascular dysfunctions by modifying the structure and function of essential membrane proteins [40, 41] and enzymes. As a result, treatment with the methanol extract of *C. pentandra* could prevent cell dysfunctions and tissue damage associated with the oxidation of proteins.

All results obtained so far have shown that the extracts from the bark of the trunk of *Ceiba pentandra* have antioxidant properties. These effects may be attributable to the presence of certain secondary metabolites. The reason being that, polyphenols, a heterogeneous group of phenolic compounds (flavonoids, anthocyanins, phenolic acids, etc.) have an ideal chemical structure for trapping free radicals [42]. The evaluation of the polyphenol and flavonoid contents shows that the methanol extract contains nearly twice as much polyphenols and flavonoids than the aqueous extract. Besides, the LC-MS shows the presence of compounds that could enable them to be good antioxidant candidates. Although none of the identified compounds have been shown to possess antioxidant activities, their derivatives are well known antioxidants. Indeed, many isoflavones [43, 44] and trimethoxyphenol [45, 46] derivatives have exhibited antioxidant activities. The presence and the concentration of these substances in both extracts could explain, on one hand, why these extracts both have antioxidant activities, and on the other hand, why the methanol extract has a scavenging activity greater than that of the aqueous extract.

Type 2 diabetes is a progressive disease whose first step is the loss of postprandial glycemic control [47]. The resulting postprandial hyperglycemia is an independent risk factor of cardiovascular diseases and neurological dysfunctions. One of the mechanisms through which postprandial hyperglycemia induces vascular damages is oxidative stress [48]. Thus, postprandial glucose control is important not only for regulating glycemia but also for helping mitigate diabetes exacerbation and complications.

Several studies have shown that polyphenols have hypoglycemic effects at different levels: the protection of pancreatic *β*-cells against glucotoxicity, inhibition of carbohydrate digestion by inhibition of *α*-glucosidases, inhibition of intestinal glucose uptake, inhibition of glucose production by the liver, improvement of glucose uptake in peripheral tissues, and inhibition of AGE formation [49]. Fofié et al. [14] have shown that extracts from *Ceiba pentandra* prevent glucose production from the liver and enhance glucose consumption. Moreover, AGE formation was shown to be prevented by *C. pentandra* extracts [15]. As these mechanisms were already investigated, we further elucidated the property of these extracts to inhibit *α*-glucosidases.

The *α*-amylase inhibition test shows that the methanol extract was three times more effective at inhibiting this enzyme than acarbose and about 9 times more effective than the aqueous extract. The evaluation of the inhibition mode shows that the methanol extract has a competitive mode. In this case, the extract and the substrate (starch) have the same binding site on *α*-amylase and they compete for this site. The implication of this mode of inhibition is that an excess of polysaccharide in food can significantly reduce its action. Therefore, its dose has to be calibrated depending on the quantity of food to be absorbed. As for the aqueous extract, the inhibition mode was a noncompetitive mixed one. In this case, the inhibitor found in the extract will bind to the enzyme whether or not the enzyme has already bound the starch molecules. However, this effect can be partially overcome by a high substrate concentration. Inhibition of *α*-glucosidase shows that the methanol extract has an efficiency 1.5 times greater than that of the aqueous extract. The mode of inhibition of the methanol extract is a pure noncompetitive type meaning that the affinity of the enzyme for the inhibitor is not changed whether bound to the substrate or not. This implies that, no matter the quantity of carbohydrate absorbed, the effect of the inhibitor would not be affected. The inhibition mode of the aqueous extract is a mixed noncompetitive type. Most of the carbohydrates we eat are in the form of poly- and disaccharides. These require prior digestion into monosaccharides in order to be absorbed through the intestinal epithelium. Inhibition of this digestion is ensured by the extracts therefore making it possible to reduce the influx of glucose and fructose into the hepatic portal vein and thus limiting the amplitude of postprandial hyperglycemic peaks and the risks of diabetes onset and its complications [50]. This inhibition of digestive enzymes by the extracts may be attributable to the presence of isoflavones as many have been shown to possess inhibitory properties on *α*-amylase and *α*-glucosidase activity [51, 52].

## 5. Conclusion

Results of the present work show that the aqueous and methanol extracts from the bark of *Ceiba pentandra* have scavenging properties which underlie their capacity to prevent the oxidation of biological molecules and thus maintain cell and tissue integrity. This ability is probably related to the presence of the phenolic compounds they contain. In addition, these extracts prevent postprandial hyperglycemia in part by inhibiting the activity of intestinal enzymes involved in the digestion of carbohydrates. These data thus suggest that extracts of *Ceiba pentandra* are excellent therapeutic candidates for the remediation of diabetes and its associated complications.

## Figures and Tables

**Table 1:** Summary of the antioxidant and carbohydrate digestive enzyme inhibitory effects of *C. pentandra* extracts.

	Antioxidant activities								Carbohydrate digestive enzymes			
	Superoxide anion		Hydrogen peroxide		AAPH protein		H_2_O_2_ protein		*α*-Amylase		*α*-Glucosidase	
	IC_50_	EI	IC_50_	EI	IC_50_	EI	IC_50_	EI	IC_50_	EI	IC_50_	EI
Methanol extract	51.81	1.3	44.84	1.5	39.26	1.09	120.6	0.44	6.15	14.93	76.61	1.14
Aqueous extract	34.26	1.26	1.78	12.07	97.95	0.28	140.4	0.04	54.52	1.62	86.49	0.78
Gallic acid	55.66	1.43			90.17	0.64	29.9	1.74				
Ascorbic acid			13.84	4.77								
Acarbose									20.97	4.03	8.49	11.76
*F*	2.77		6.13		33.65		74.23		64.54		270.40	
*p*	0.0384		0.0058		<0.0001		<0.0001		<0.0001		<0.0001	

IC_50_: concentration of the tested substance able to inhibit 50% of the activity; EI: efficiency index, calculated as maximal activity/IC_50_. The statistical analysis was performed with nonlinear regression and the best fit parameters (logIC_50_ and top) were compared.

## Data Availability

The data used and analyzed in this study are available from the corresponding author on reasonable request.

## References

[1] International Diabetes Federation (2019). *IDF Diabetes Atlas*.

[2] Gerich J. (2013). Pathogenesis and management of postprandial hyperglycemia: role of incretin-based therapies. *International Journal of General Medicine*.

[3] Numao S. (2016). A single bout of exercise and postprandial hyperglycemia caused by high-fat diet. *The Journal of Physical Fitness and Sports Medicine*.

[4] Ceriello A. (2005). Postprandial hyperglycemia and diabetes complications: is it time to treat?. *Diabetes*.

[5] Rangel É. B., Rodrigues C. O., de Sá J. R. (2019). Micro- and macrovascular complications in diabetes mellitus: preclinical and clinical studies. *Journal Diabetes Research*.

[6] Chawla A., Chawla R., Jaggi S. (2016). Microvasular and macrovascular complications in diabetes mellitus: distinct or continuum?. *Indian Journal of Endocrinology and Metabolism*.

[7] Yan L. J. (2014). Pathogenesis of chronic hyperglycemia: from reductive stress to oxidative stress. *Journal Diabetes Research*.

[8] Brownlee M. (2001). Biochemistry and molecular cell biology of diabetic complications. *Nature*.

[9] Wu Y., Tang L., Chen B. (2014). Oxidative stress: implications for the development of diabetic retinopathy and antioxidant therapeutic perspectives. *Oxidative Medicine and Cellular Longevity*.

[10] Sakaguchi K., Kasuga M. (2007). Adverse effects of alpha-glucosidase inhibitors. *Nihon Rinsho*.

[11] Martínez L., Ros G., Nieto G. (2018). Hydroxytyrosol: health benefits and use as functional ingredient in meat. *Medicine*.

[12] Heim K. E., Tagliaferro A. R., Bobilya D. J. (2002). Flavonoid antioxidants: chemistry, metabolism and structure-activity relationships. *The Journal of Nutritional Biochemistry*.

[13] Xiao J. B., Hogger P. (2015). Dietary polyphenols and type 2 diabetes: current insights and future perspectives. *Current Medicinal Chemistry*.

[14] Fofie C. K., Wansi S. L., Nguelefack-Mbuyo E. P. (2014). In vitro anti-hyperglycemic and antioxidant properties of extracts from the stem bark of *Ceiba pentandra*. *Journal of Complementary and Integrative Medicine*.

[15] Fofie K. S., Nguelefack-mbuyo K. A., Kamble B., Chauhan N., Singh V., Nguelefack T. B. (2018). Insulin sensitizing effect as possible mechanism of the antidiabetic properties of the methanol and the aqueous extracts from the trunk bark of *Ceiba pentandra*. *Diabetes Updates*.

[16] Fofié C. K., Nguelefack-Mbuyo E. P., Tsabang N., Kamanyi A., Nguelefack T. B. (2018). Hypoglycemic properties of the aqueous extract from the stem bark of *Ceiba pentandra* in dexamethasone-induced insulin resistant rats. *Evidence-based Complementary and Alternative Medicine*.

[17] Fofie C. K., Katekhaye S., Borse S. (2019). Antidiabetic properties of aqueous and methanol extracts from the trunk bark of *Ceiba pentandra* in type 2 diabetic rat. *Cell Biochemistry*.

[18] McDonald S., Prenzler P. D., Antolovich M., Robards K. (2001). Phenolic content and antioxidant activity of olive extracts. *Food Chemistry*.

[19] Chang C. C., Yang M. H., Wen H. M., Chern J. C. (2002). Estimation of total flavonoid content in propolis by two complementary colorimetric methods. *Journal of Food and Drug Analysis*.

[20] Robak J., Gryglewski R. J. (1988). Flavonoids are scavengers of superoxide anions. *Biochemical Pharmacology*.

[21] Ruch R. J., Cheng S. J., Klaunig J. E. (1989). Prevention of cytotoxicity and inhibition of intercellular communication by antioxidant catechins isolated from Chinese green tea. *Carcinogenesis*.

[22] Di Simplicio P., Cheeseman K. H., Slater T. F. (1991). The reactivity of the SH group of bovine serum albumin with free radicals. *Free Radical Research Communications*.

[23] McCue P. P., Shetty K. (2004). Inhibitory effects of rosmarinic acid extracts on porcine pancreatic amylase in vitro. *Asia Pacific Journal of Clinical Nutrition*.

[24] Ali H., Houghton P. J., Soumyanath A. (2006). *α*-Amylase inhibitory activity of some Malaysian plants used to treat diabetes; with particular reference to *Phyllanthus amarus*. *Journal of Ethnopharmacology*.

[25] Saboury A. A. (2009). Enzyme inhibition and activation: a general theory. *Journal of the Iranian Chemical Society*.

[26] Kim Y. M., Jeong Y. K., Wang M. H., Lee W. Y., Rhee H. I. (2005). Inhibitory effect of pine extract on *α*-glucosidase activity and postprandial hyperglycemia. *Nutrition*.

[27] Rao K. V., Sreeramulu K., Gunasekar D., Ramesh D. (1993). Two new sesquiterpene lactones from *Ceiba pentandra*. *Journal of Natural Products*.

[28] Kishore P. H., Reddy M. V. B., Gunasekar D., Caux C., Bodo B. (2003). A new naphthoquinone from *Ceiba pentandra*. *Journal of Asian Natural Products Research*.

[29] Faizi S., Zikr-Ur-Rehman S., Versiani M. A. (2011). Shamiminol: a new aromatic glycoside from the stem bark of *Bombax ceiba*. *Natural Product Communications*.

[30] Joshi K. R., Devkota H. P., Yahara S. (2014). Simalin A and B: two new aromatic compounds from the stem bark of *Bombax ceiba*. *Phytochemistry Letters*.

[31] Ueda-Wakagi M., Mukai R., Fuse N., Mizushina Y., Ashida H. (2015). 3-O-acyl-epicatechins increase glucose uptake activity and GLUT4 translocation through activation of PI3K signaling in skeletal muscle cells. *International Journal of Molecular Sciences*.

[32] Noreen Y., el-Seedi H., Perera P., Bohlin L. (1998). Two new isoflavones from *Ceiba pentandra* and their effect on cyclooxygenase-catalyzed prostaglandin biosynthesis. *Journal of Natural Products*.

[33] Refaat J., Desoky S. Y., Ramadan M. A., Kamel M. S. (2013). Bombacaceae: a phytochemical review. *Pharmaceutical Biology*.

[34] Anwar F., Rashid U., Shahid S. A., Nadeem M. (2014). Physicochemical and antioxidant characteristics of kapok (*Ceiba pentandra* Gaertn.) seed oil. *Journal of the American Oil Chemists' Society*.

[35] Pavithra K., Vadivukkarasi S. (2015). Evaluation of free radical scavenging activity of various extracts of leaves from *Kedrostis foetidissima* (Jacq.) Cogn.. *Food Science and Human Wellness*.

[36] Nimse S. B., Pal D. (2015). Free radicals, natural antioxidants, and their reaction mechanisms. *RSC Advances*.

[37] Wang Y., Branicky R., Noë A., Hekimi S. (2018). Superoxide dismutases: dual roles in controlling ROS damage and regulating ROS signaling. *The Journal of Cell Biology*.

[38] Li W., Wu Z., Ma Q. (2014). Hyperglycemia regulates TXNIP/TRX/ROS axis via p38 MAPK and ERK pathways in pancreatic cancer. *Current Cancer Drug Targets*.

[39] Pratt D. A., Tallman K. A., Porter N. A. (2011). Free radical oxidation of polyunsaturated lipids: new mechanistic insights and the development of peroxyl radical clocks. *Accounts of Chemical Research*.

[40] Niedowicz D. M., Daleke D. L. (2005). The role of oxidative stress in diabetic complications. *Cell Biochemistry and Biophysics*.

[41] Ramana K. V., Srivastava S., Singhal S. S. (2017). Lipid peroxidation products in human health and disease 2016. *Oxidative Medicine and Cellular Longevity*.

[42] Kadiri O. (2017). A review on the status of the phenolic compounds and antioxidant capacity of the flour: effects of cereal processing. *International Journal of Food Properties*.

[43] Rüfer C. E., Kulling S. E. (2006). Antioxidant activity of isoflavones and their major metabolites using different in vitro assays. *Journal of Agricultural and Food Chemistry*.

[44] Yoon G.-A., Park S. (2014). Antioxidant action of soy isoflavones on oxidative stress and antioxidant enzyme activities in exercised rats. *Nutrition Research and Practice*.

[45] Matos M. A. R., Miranda M. S., Morais V. M. F. (2008). 3,4,5-Trimethoxyphenol: a combined experimental and theoretical thermochemical investigation of its antioxidant capacity. *The Journal of Chemical Thermodynamics*.

[46] Falah S., Katayama T., Suzuki T. (2008). Chemical constituents from *Gmelina arborea* bark and their antioxidant activity. *Journal of Wood Science*.

[47] Monnier L., Colette C., Dunseath G. J., Owens D. R. (2007). The loss of postprandial glycemic control precedes stepwise deterioration of fasting with worsening diabetes. *Diabetes Care*.

[48] Ceriello A. (2009). Postprandial hyperglycemia and cardiovascular disease: is the HEART2D study the answer?. *Diabetes Care*.

[49] Hanhineva K., Törrönen R., Bondia-Pons I. (2010). Impact of dietary polyphenols on carbohydrate metabolism. *International Journal of Molecular Sciences*.

[50] Kumar S., Narwal S., Kumar V., Prakash O. (2011). *α*-Glucosidase inhibitors from plants: a natural approach to treat diabetes. *Pharmacognosy Reviews*.

[51] Wu C., Shen J., He P. (2012). The *α*-glucosidase inhibiting isoflavones isolated from *Belamcanda chinensis* leaf extract. *Records of Natural Products*.

[52] Kim D. H., Yang W. T., Cho K. M., Lee J. H. (2020). Comparative analysis of isoflavone aglycones using microwave-assisted acid hydrolysis from soybean organs at different growth times and screening for their digestive enzyme inhibition and antioxidant properties. *Food Chemistry*.

